# miRNA 548a-3p as biomarker of NEDA-3 at 2 years in multiple sclerosis patients treated with fingolimod

**DOI:** 10.1186/s12974-023-02811-z

**Published:** 2023-05-30

**Authors:** Alicia Gonzalez-Martinez, Rohit Patel, Brian C. Healy, Hrishikesh Lokhande, Anu Paul, Shrishti Saxena, Mariann Polgar-Turcsanyi, Howard L. Weiner, Tanuja Chitnis

**Affiliations:** 1 Department of Neurology, Translational Neuroimmunology Research Center (TNRC), Ann Romney Center for Neurologic Diseases (ARCND), Brigham and Women’s Hospital, 60 Fenwood Road, 9002K, Boston, MA 02115 USA; 2 Harvard Medical School, Boston, MA 02115 USA; 3 Department of Neurology, Brigham MS Center, Brigham and Women’s Hospital, Boston, MA 02115 USA

**Keywords:** miRNA, Long-term, Disability, EDSS, miR-548a-3p, NEDA-3, Multiple sclerosis, Biomarker, RRMS

## Abstract

**Background:**

Multiple sclerosis (MS) is a disabling autoimmune demyelinating disorder affecting young people and causing significant disability. In the last decade, different microRNA (miRNA) expression patterns have been associated to several treatment response therapies such as interferon and glatiramer acetate. Nowadays, there is increasing interest in the potential role of miRNA as treatment response biomarkers to the most recent oral and intravenous treatments. In this study, we aimed to evaluate serum miRNAs as biomarkers of No Evidence of Disease Activity (NEDA-3) at 2 years in patients with relapsing remitting MS (RRMS) treated with fingolimod.

**Main body:**

A Discovery cohort of 31 RRMS patients treated with fingolimod were identified from the CLIMB study and classified as No Evidence of Disease Activity (NEDA-3) or Evidence of Disease Activity (EDA-3) after 2 years on treatment. Levels of miRNA expression were measured at 6 months using human serum miRNA panels and compared in EDA-3 and NEDA-3 groups using the Wilcoxon rank sum test. A set of differentially expressed miRNA was further validated in an independent cohort of 22 fingolimod-treated patients. We found that 548a-3p serum levels were higher levels in fingolimod-treated patients classified as NEDA-3, compared to the EDA-3 group in both the Discovery (*n* = 31; *p* = 0.04) and Validation (*n* = 22; *p* = 0.03) cohorts 6 months after treatment initiation; miR-548a-3p provided an AUC of 0.882 discriminating patients with NEDA-3 at 2 years in the Validation cohort.

**Conclusion:**

Our results show differences in miR-548a-3p expression at 6 months after fingolimod start in patients with MS with NEDA-3 at 2 years. These results provide class III evidence of the use of miR-548a-3p as biomarker of NEDA-3 in patients with fingolimod.

**Supplementary Information:**

The online version contains supplementary material available at 10.1186/s12974-023-02811-z.

## Background

Multiple sclerosis (MS) is an autoimmune demyelinating disease of the CNS [1]. Currently, cerebral MRI is the main tool used as a biomarker to diagnose and monitor MS activity [2]. There is increasing interest in the identification of blood biomarkers to monitor disease activity and treatment response in MS.

MicroRNA (miRNA) are small non-coding RNAs that regulate the expression of genes at the posttranscriptional level by binding to complementary sequences in the 3′ or 5′ untranslated region (UTR) of the target Messenger RNA [3]. Circulating miRNAs can be detected in blood and are stable following extended storage, freeze–thawing, and extreme pH [4, 5]. Their stability, along with the development of sensitive methods for their detection and quantification [6] makes circulating miRNAs ideal candidates for biomarkers. Previous studies performed in our Comprehensive Longitudinal Investigation of Multiple Sclerosis at the Brigham and Women’s Hospital (CLIMB)-cohort have identified different miRNA expression patterns associated with MS type, disease progression and MRI features, being considered potential biomarkers of disease and disability [7–9]. In addition, there is growing evidence of the role of miRNA as a biomarkers of treatment response in MS [10, 11].

In the present study, we investigated the role of serum miRNA as predictors of No Evidence of Disease Activity (NEDA-3) at 2 years in patients treated with fingolimod. We hypothesize that miRNA expression levels could act as biomarkers of sustained NEDA-3 at 2 years in patients under fingolimod treatment.

## Methods

### Study population

The aim of this study was to identify circulating miRNAs and evaluate their role as biomarkers of NEDA-3 status at 2 years in MS patients on fingolimod treatment (Class III level of evidence). Samples from patients with MS were obtained from the CLIMB Study. CLIMB is an ongoing longitudinal cohort study that follows more than 2000 patients with clinical examinations, MRI, and blood sampling done on a yearly basis. For the present study, we analyzed the serum miRNA of patients diagnosed with MS as defined by the McDonald criteria 6 to 12 months after fingolimod treatment start [1] regarding NEDA-3 status. Blood samples were collected between 10:00 am and 17:00 pm, following oral fingolimod intake as prescribed by the neurologist in charge in real-life clinical practice. Patients achieving NEDA-3 were patients whose EDSS remains unchanged, have no relapse nor disease activity in MRI during the 2 years follow-up period. Patients who had relapses, brain MRI activity, or sustained disability worsening were classified as showing evidence of disease activity (EDA-3).

### Standard protocol approvals and consent forms

Written informed consent was obtained from all patients included in the CLIMB Study. This study was approved by the Mass General Brigham Humans Research Committee.

### Study design

**Fig. 1:**
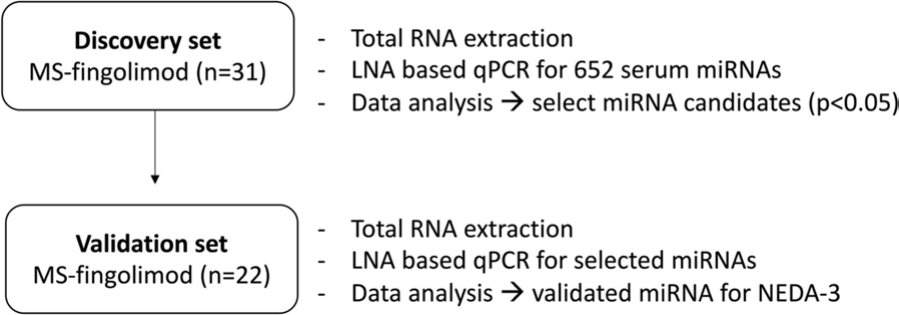
Flowchart. Flowchart showing the study design. *qPCR* quantitative PCR, *miRNA* microRNA, *MS* multiple sclerosis

The study was performed in two phases: discovery and validation phase (Fig. 1). In the discovery phase, 652 miRNAs were measured in fingolimod-treated patients (*n* = 31). We compared between patients with NEDA-3 versus EDA-3 status at 2 years.

Based on the rank order from the discovery phase and availability of the miRNA in the second customized panel, a subset of miRNA was measured in the validation set of fingolimod-treated patients (*n* = 22). The demographic characteristics of the groups are shown in Table 1.

**Table 1:** Demographic and clinical characteristics of the patients included in the study

	Discovery set		
	All sample	NEDA 3	EDA-3
No. of participants	31	19	12
Mean age (SD)	40.71 (9.81)	41.79 (10.17)	39 (9.38)
Females %	71	73.7	66.7
Mean EDSS score (SD)	1.52 (1.17)	1.5 (1.07)	1.54 (1.36)
Mean relapses in the previous 2 years	0.65 (1.54)	0.74 (1.85)	0.5 (0.9)

	Validation set		
	All sample	NEDA 3	EDA-3
No. of participants	22	17	5
Mean age (SD)	44.36 (11.09)	42.65 (10.89)	50.18 (10.79)
Females %	81.8	82.4	80
Mean EDSS score (SD)	1.82 (1.28)	1.38 (1.11)	3.3 (0.27)
Mean relapses in the previous 2 years	0.45 (0.67)	0.53 (0.72)	0.2 (0.45)

### Samples and methods

Blood samples were collected in glass red-top serum vacutainer tubes without additives (BD, Franklin Lakes, NJ); serum tubes were kept at room temperature for 30 to 60 min. Each sample was centrifuged at 2000 rpm for 10 min to separate serum and then stored at − 70 °C until RNA extraction. Serum was frozen within 2 h of the blood draw.

### Quantification of miRNA

#### RNA isolation

RNA was isolated using the miRcury kit (Exiqon, Woburn, MA) and converted to complementary DNA using a cDNA synthesis kit from Exiqon following the manufacturer’s instructions. Prepared complementary DNAs were stored at − 20 °C until use.

#### Quantitative reverse transcriptase PCR

Locked nucleic acid (LNA) SYBR green-based real-time PCR Human Panel I and II (Exiqon) containing 652 miRNAs were used for profiling in the discovery phase. Normalization was performed using NormFinder. The best normalizer was found to be the average of assays detected in all samples. All data were normalized to the average of assays detected in all samples. The formula used to calculate the normalized Cq values (dCq) is: dCq = average assay Cq − assay Cq. The Cq value represents the number of PCR cycles required for the miRNA signal to reach a certain threshold, and is used as a measure of the amount of RNA present in the sample. A positive dCq value indicates that the target of interest is more expressed in the sample in question than in the reference sample, while a negative dCq value indicates less expression. The dCq value is often used to calculate the relative expression of a target in different samples by normalizing the miRNA expression values to a reference.

#### miRNA targets and pathways

We performed in silico miRNA target analysis and enrichment analysis using DIANA-miRPath v3.0 [12]. To identify the target genes we used TarBasev7.0. To perform pathway analysis we used Kyoto Encyclopedia of Genes Genomes (KEGG) [13]. In a similar way Gene Ontology (GO) analysis was also performed to find the enriched categories.

### T cell isolation and transfection

We performed an exploratory functional analysis to evaluate the effect of the miRNA on T regulatory (reg) cells, by using miRNA mimics and inhibitors, measuring the percentage of CD4+CD25+Foxp3 (Treg) population and cytokine production. Pan T cells from PBMCs of healthy control donor were isolated using Pan T cell isolation kit (Miltenyi Biotec, Bergisch Gladbach, Germany). Approximately 75 × 10 [3] cells were seeded per well in a U-bottom 96-well plate followed by transfection with miRNA-548a-3p mimic or inhibitor or negative control (Qiagen, Hilden, Germany). Briefly, mimic (20 nM) or inhibitor (50 nM) was mixed with HiPerfect transfection reagent to prepare transfection complex and followed the manufacturer’s protocol for transfection of suspension cells. Cells were grown for 24 h at 37 °C in incubator with 5% CO_2_. Transfection efficiency was determined by analyzing the expression of miRNA 548a-3p by qRT-PCR as discussed above. U6snRNA was used as loading control for miRNA expression.

### FACS analysis of T cell-associated markers

Approximately 75 × 10^3^ cells were transfected (*n* = 6) with miRNA 548a-3p mimic or inhibitor or negative control in a U-bottom 96-well plate and incubated for 24 h in cell culture medium containing 10% heat inactivated FBS. This was followed by replacing the medium with fresh culture medium and induction with anti-CD3/28 (StemCell, Vancouver, Canada) for additional 24 h. After the incubation was over, cells were stained with anti-CD4 APC/Fire 750, anti-CD25 Pacific blue and anti-FoxP3 FITC, anti IL17 Percp/Cy5.5, anti IFNγ PE/Cy7 (BioLegend, San Diego, CA, USA), anti IL10 BV711 (BD Biosciences, San Jose, CA, USA), anti Akt2 AF647 (R&D System, NE Minneapolis, MN, USA) and fix viability dye (Invitrogen, Waltham, MA, USA) as previously described [14]. After staining was done, cells were resuspended in FACS buffer. Cell samples were then run in LSRFortessa™ Cell Analyzer (BD Biosciences) and data were analyzed using FlowJo software.

### Statistical analysis

Values are listed as mean ± SD or range. Patients achieving NEDA-3 at 2 years in the discovery set were compared to patients with EDA-3 for each miRNA using a Wilcoxon rank sum test. A Wilcoxon rank sum test was used so that participants with miRNA levels below the limit of detection (missing or undetected values) could contribute to the analysis. Undetected expression values were assigned a value lower than the smallest observed value from all participants. All miRNAs were then rank ordered based on the Wilcoxon rank sum test *p* value. An adjusted *p* value from proportional odds model adjusting for age, sex and EDSS at baseline was also obtained. To select miRNAs for the validation phase, we chose up to 5 miRNAs based on (1) the Wilcoxon rank sum test *p* value and (2) the presence at a second pre-customized human miRNA panel, with the requirement that the miRNA was expressed by at least 50% of the participants in at least one of the groups and the *p* value was less than 0.05. In the validation phase, each miRNA identified based on the discovery phase was compared between the NEDA-3 and EDA-3 patients using the same approach as in the discovery phase.

The receiver operating characteristic curve is a graphical approach for investigating the sensitivity and specificity at all possible cutoff values for a predictor, and the area under the receiver operating characteristic curve (AUC) provides an estimate of the miRNA’s ability to discriminate the groups compared [15]. The AUCs were calculated for each miRNA. We also compared the miRNA expression level between the groups using a proportional odds model to adjust for age and sex. The proportional odds model is a generalization of the Wilcoxon rank sum test that allows adjustment for other variables [16].

A miRNA was defined as significantly differentially expressed in the validation phase if it was expressed by at least 50% of the participants in at least one of the groups, the Wilcoxon test *p* value was less than 0.05, and the same direction of expression (up or downregulated) was observed in both phases.

Finally, we studied the correlation between lymphocyte counts and NEDA-3 at 2 years and between lymphocyte counts and miRNA expression using Wilcoxon test or Spearman correlation depending on the nature of the variables.

Statistical analysis was completed using the statistical packages R (www.r-project.org) and Stata/IC version 17 (www.sata.com).

### Data availability

Anonymized data not published within this article will be made available by request from any qualified investigator. The principal author had full access to those data and has maintained the right to publish all data independent of any third party. This study has been approved by our hospital’s Ethics Committee and follows the STARD guidelines (Additional file 1).

## Results

We included a total of 53 patients (Fig. 1), mean age 42.2 (SD: 12.20) years, 75.47% females, mean EDSS 1.64 (SD: 1.15), 0.6 (SD: 1.5) relapses in the previous 2 years (Table 1). There were 6 treatment naïve patients, 3 in the first cohort and 3 in the second cohort). Mean time from treatment start to blood sampling was 8.4 (SD 2.8) months. Three EDA-3 patients were treated with steroids between 6 and 12 months from fingolimod start in a range between 2 weeks and 10 months before blood sampling.

In the discovery set 31 patients were studied, 40.71 (9.81) years old, 71% women, mean EDSS 1.52 (SD: 1.17), 0.5 (SD: 0.9) relapses in the previous 2 years.

In the validation set, 22 patients were studied, 44.36 (11.09) years old, 81.8% women, mean EDSS 1.82 (1.28), 0.45 (SD: 0.67) relapses in the previous 2 years (Table 2).

**Table 2:** Validated serum miRNA differentially expressed between patients achieving NEDA-3 and EDA-3 at 2 years

miRNA	Discovery cohort (*n* = 31)			Validation cohort (*n* = 22)		
	Mean expression			Mean expression		
	NEDA-3	EDA-3	Adjusted *p* value*	NEDA-3	EDA-3	Adjusted *p* value*
Hsa-mir-487b.3p	− 8.540	− 9.691	< 0.001	− 6.404	− 7.354	0.135
Hsa-mir-380.3p	− 10.808	− 11.467	0.008	− 9.573	− 9.225	0.761
Hsa-mir-376b.3p	− 7.446	− 8.125	0.011	− 6.670	− 7.025	0.348
Hsa-mir-548a-3p	− 7.223	− 8.176	0.044	− 5.585	− 7.199	0.031
Hsa-mir-301b	− 8.375	− 8.499	0.024	− 7.378	− 7.550	0.252

Top 5 miRNA expressed in both cohorts

*miRNA* microRNA, *MS* multiple sclerosis

*Adjusted *p* value from proportional odds model adjusting for age, sex and EDSS at baseline

**Fig. 2:**
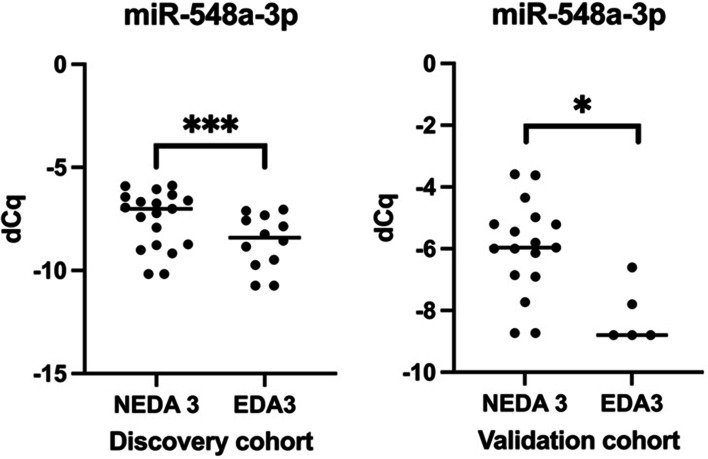
miR-548a-3p expression levels in the Discovery and Validation cohort. dCq: delta Cq. Observations below the limit of detection are shown on the graph subtracting 1 to the smallest value. A higher value thus indicates that the microRNA is more abundant in the particular sample. *NEDA-3* No Evidence of Disease Activity, *EDA-3* Evidence of Disease Activity. *p < 0.05; **p < 0.01; ***p < 0.001; ****p < 0.0001

A total of 24 miRNA were differentially expressed between patients with MS under fingolimod treatment regarding NEDA-3 at 2 years. After filtering using the selection criteria, 5 miRNAs were chosen for further validation. We found that miR-548a-3p was significantly differentially expressed in MS patients with sustained NEDA-3 at 2 years compared to EDA-3 regarding the Wilcoxon rank sum test *p* value and after adjusting for sex, age and EDSS at baseline (Fig. 2).

**Fig. 3:**
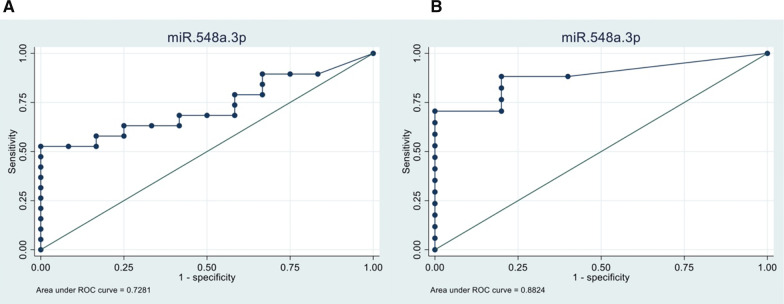
ROC curves for 548a-3p expression levels in the Discovery (Cohort 1) and Validation cohort (Cohort 2). ROC curve for NEDA-3 vs EDA-3 regarding miR-548a-3p in the Discovery (**A**) and Validation (**B**) cohort

In the Validation cohort, we found that miR-548a-3p provided an AUC (0.882) discriminating patients with NEDA-3 at 2 years. A graphic representation of the ROC curves is included in Fig. 3.

### Immune associations

We evaluated associations with lymphocyte counts. There was no difference in lymphocyte counts at 6–12 months from fingolimod onset in the EDA-3 and NEDA-3 groups at 2 years (Wilcoxon *p* value = 0.98). Moreover, there was no correlation between the lymphopenia grade 1 or 2 at 6–12 months and NEDA-3 at 2 years (*p* value = 0.36) nor lymphopenia grade 3 or 4 and NEDA-3 at 2 years (*p* value = 0.7), included in Additional file 2: Table S1.

In addition, we explored the correlation between lymphopenia and miRNA expression and we did not find a correlation between the lymphocyte counts and miR-548a-3p in the discovery set (Wilcoxon *p* value = 0.84) nor the validation set (Wilcoxon *p* value = 0.67).

Regarding the exploratory functional analysis, we successfully isolated T cells from the donors and effectively transfected them with miR-548a-3p mimic or inhibitor as shown in the qPCR results included in Additional file 2: Fig. S1.

The output measurements including the percentage of CD4+CD25+Foxp3 (Treg) population and cytokine production including IL-17, IFNg and IL-10, and did not reveal any significant difference between the groups with miR-548a-3p mimics or inhibitors compared to the control group.

### Pathways and target genes

There were 247 genes targeted by miR-548a-3p using Tarbasev7.0. We identified two pathways non-alcoholic fatty liver disease (nafld/hsa04932) and viral carcinogenesis (hsa05203) in which there were 11 and 7 genes, enriched, respectively.

Using GO analysis many categories were enriched. Among them the top three enriched pathways were organelle (150), molecular function (227) and biological processes (222).

**Fig. 4:**
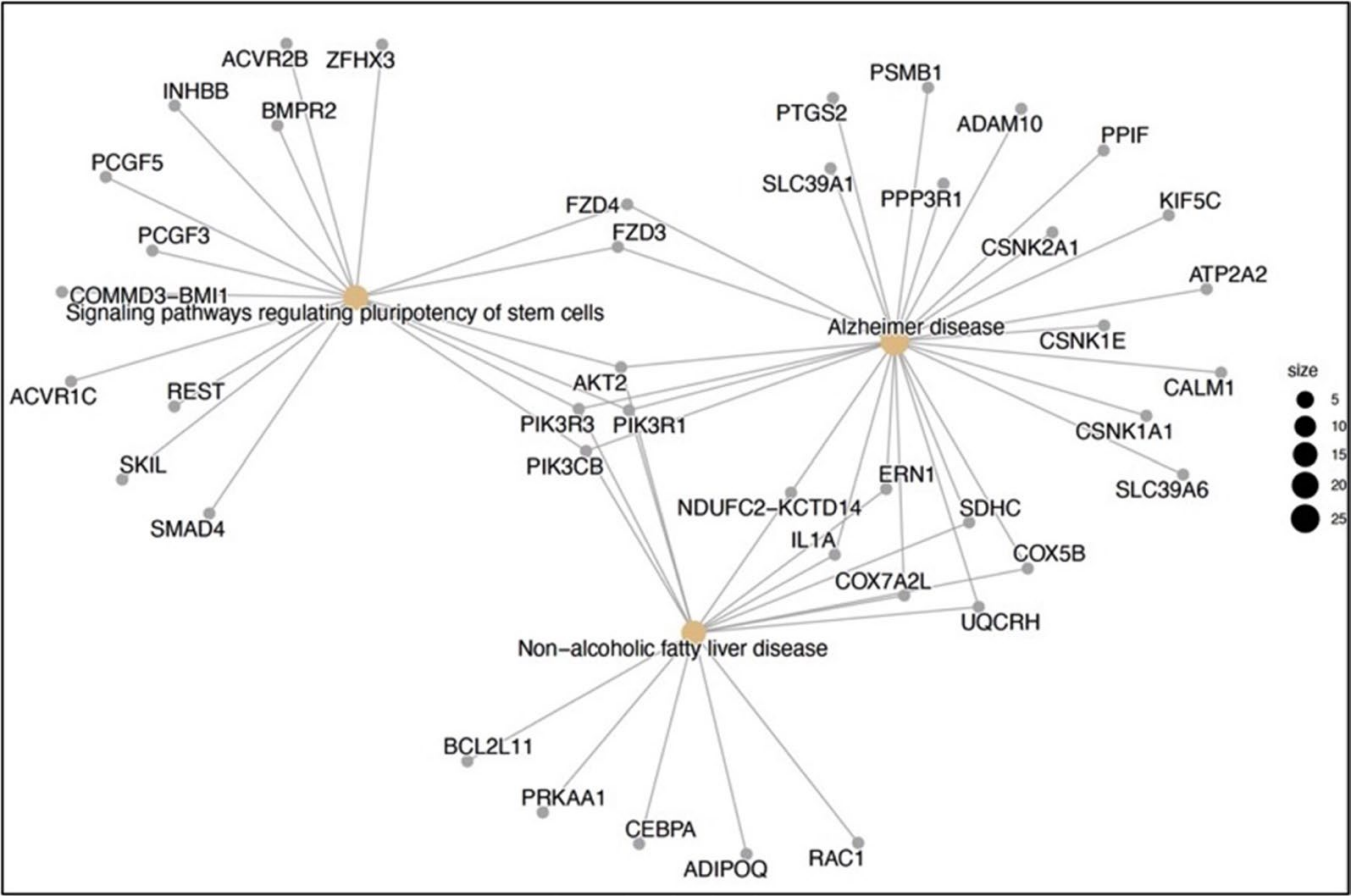
Predicted target genes

In the enrichment analysis AKT2, PIK3R1, PIK3R3 and PIK3CB were the main target genes regulated by miR-548a-3p (Fig. 4).

The exploratory functional analysis revealed that miR-548-3p mimic alone significantly increased mean fluorescence intensity (MFI) of Treg although it did not change the percentage of Treg population, cytokine production or AKT2 expression in T cells (Additional file 2: Fig. S1).

## Conclusions

We have identified and validated the presence of changes in an easily accessible serum miR-548a-3p among patients treated with fingolimod achieving NEDA-3 and EDA-3 at 2 years in two independent cohorts.

Previous studies have demonstrated the role of miRNA as biomarkers of disease in MS [6]. A classical approach based on a two-step strategy using a Discovery and Validation cohort is widely used for disease and treatment response biomarker search [7]. In our study, we validated one miRNA, miR-548a-3p, as a biomarker of treatment response in patients with MS under fingolimod therapy. We found that miR-548a-3p provided an AUC (0.882) in discriminating patients with NEDA-3 at 2 years in the Validation cohort. These results provide a new tool for monitoring treatment response in clinical practice.

Previous studies have shown that miR-548a-3p is dysregulated in autoimmune diseases [17–19]. miR-548a-3p has been found to inhibit the proliferation and activation of macrophage-like (pTHP-1) cells by regulating the TLR4/NF-κB signaling pathway in rheumatoid arthritis [17]. Moreover, miR-548a-3p has been involved in keratinocyte proliferation by targeting PPP3R1 and T regulatory cells, pointing out a possible implication in the pathogenesis of psoriasis, a T cell-mediated autoimmune disease [18]. More recently, miR-548a-3p has been found to be significantly upregulated in acute graft-versus-host disease patients, suggesting its role as a potential noninvasive biomarkers in this disease [19]. However, our study is the first one to identify miR-548a-3p as treatment response biomarker in MS patients under fingolimod treatment.

Beyond the role of miR-548a-3p as treatment response biomarker in MS patients treated with fingolimod, we performed an enrichment analysis to identify potential pathways and targets. In the enrichment analysis AKT2, PIK3R1, PIK3R3 and PIK3CB were the main target genes regulated by miR-548a-3p. Some studies have pointed out the link between miR-548a-3p and SP1 therapies. *Akt2* is one of the genes involved in the intracellular signaling cascade connected to S1P1 and CXCR4, which has been found to be a predictive target gene of miR-548a-3p according to our enrichment analysis. Moreover, miR-548a-3p was also found to be associated with protein kinase cascades, lymphocyte proliferation, and apoptosis in human lymphoblastoid cell lines [20]. In addition, *Akt2* has been shown to be regulated by fingolimod in studies performed in animal MOG-induced EAE models [21]. However, according to our exploratory functional analysis, miR-548a-3p alone does not have a great effect on Treg number nor cytokine production through Akt2 expression, suggesting that miR-548a-3p main effect might be driven by regulation of these other targets (PIK3R1, PIK3R3 and PIK3CB), other cell types and/or by effect on other compartments such as the central nervous system which will need further attention. Previously reported roles of miRNA included in Validation cohort are shown in Additional file 2: Table S2 [17–19, 22–25].

Moreover, we explored the association between total lymphocyte counts and miRNA expression. We did not find any correlation between lymphocyte counts nor lymphopenia levels 1–2 or 3–4 and miR-548a-3p expression. These results may point out that the source of circulating miR-548a-3p could not only be cells, but also micro-vesicles, exosomes, or apoptotic bodies in which miRNA is packaged [26, 27].

Moreover, there was no correlation between total lymphocyte counts and NEDA-3 at 2 years. Our results are in concordance with previous observations in which the lack of association has been found after 12 months in larger sample size from observational retrospective studies [28]. It has been suggested that leukocyte subpopulations might have a role in predicting response to fingolimod [29]. Future studies exploring the relationship between miR-548a-3p in different leukocyte subpopulations, may give rise of the mechanism by which miR-548a-3p participates in fingolimod response.

Among the main limitations of our study, participant samples were collected from a single MS center and there was a relatively small number of participants who contributed to each group comparison. The exact time between fingolimod oral intake and blood sampling was not available. Corticoid treatment was applied when indicated, according to treatment guidelines. Future work will require larger sample sizes to ensure that we have sufficient power to detect miRNAs with smaller effect sizes and to further study the functional role of this miRNA in MS.

As a whole, our study not only provides a new biomarker for treatment response in patients with MS under fingolimod treatment, but also opens the door for future research unraveling the functional role of miR-548a-3p in fingolimod-treated MS patients.

## Supplementary Information

**Additional file 1. **The STARD guidelines.

**Additional file 2: Table S1.** Univariate analysis of clinical variables in MS patients treated with Fingolimod regarding NEDA-3 at 2 years. **Table S2.** Previously reported roles of miRNA included in validation cohort. **Figure S1.** Exploratory functional analysis of the effect of miR-548a-3p mimic and inhibitors on T cell activation.

## Data Availability

The datasets generated and/or analyzed the current study are available from the corresponding author on reasonable request from any qualified investigator.

## Abbreviations

NEDA-3: No Evidence of Disease Activity (NEDA-3)
EDA-3: Evidence of Disease Activity (EDA-3)
RRMS: Relapsing–remitting MS
MS: Multiple sclerosis
UTR: Untranslated region
CLIMB: Comprehensive Longitudinal Investigation of Multiple Sclerosis at the Brigham and Women’s Hospital
LNA: Locked nucleic acid
KEGG: Kyoto Encyclopedia of Genes Genomes
GO: Gene Ontology
Treg: Regulatory
AUC: Area under the receiver operating characteristic curve
miRNA: MicroRNA

## References

[1] McDonald WI, Compston A, Edan G (2001). Recommended diagnostic criteria for multiple sclerosis: guidelines from the international panel on the diagnosis of multiple sclerosis. Ann Neurol.

[2] Filippi M, Rocca MA, Ciccarelli O (2016). MRI criteria for the diagnosis of multiple sclerosis: MAGNIMS consensus guidelines. Lancet Neurol.

[3] Huntzinger E, Izaurralde E (2011). Gene silencing by microRNAs: contributions of translational repression and mRNA decay. Nat Rev Genet.

[4] Mitchell PS, Parkin RK, Kroh EM (2008). Circulating microRNAs as stable blood-based markers for cancer detection. Proc Natl Acad Sci.

[5] Chen X, Ba Y, Ma L (2008). Characterization of microRNAs in serum: a novel class of biomarkers for diagnosis of cancer and other diseases. Cell Res.

[6] Guerau-de-Arellano M, Alder H, Ozer HG, Lovett-Racke A, Racke MK (2012). miRNA profiling for biomarker discovery in multiple sclerosis: from microarray to deep sequencing. J Neuroimmunol.

[7] Regev K, Paul A, Healy B (2016). Comprehensive evaluation of serum microRNAs as biomarkers in multiple sclerosis. Neurol Neuroimmunol Neuroinflamm.

[8] Regev K, Healy BC, Paul A (2018). Identification of MS-specific serum miRNAs in an international multicenter study. Neurol Neuroimmunol Neuroinflamm.

[9] Hemond CC, Healy BC, Tauhid S (2019). MRI phenotypes in MS: longitudinal changes and miRNA signatures. Neurol Neuroimmunol Neuroinflamm.

[10] Paul A, Comabella M, Gandhi R (2019). Biomarkers in multiple sclerosis. Cold Spring Harb Perspect Med.

[11] Ehtesham N, Mosallaei M, Karimzadeh MR, Moradikazerouni H, Sharifi M (2020). microRNAs: key modulators of disease-modifying therapies in multiple sclerosis.. Int Rev Immunol.

[12] Vlachos IS, Zagganas K, Paraskevopoulou MD (2015). DIANA-miRPath v3.0: deciphering microRNA function with experimental support. Nucleic Acids Res.

[13] Ogata H, Goto S, Sato K, Fujibuchi W, Bono H, Kanehisa M (1999). KEGG: Kyoto Encyclopedia of Genes and Genomes. Nucleic Acids Res.

[14] Saxena S, Lokhande H, Gombolay G, Raheja R, Rooney T, Chitnis T (2020). Identification of TNFAIP3 as relapse biomarker and potential therapeutic target for MOG antibody associated diseases. Sci Rep.

[15] Hanley JA, McNeil BJ (1982). The meaning and use of the area under a receiver operating characteristic (ROC) curve. Radiology.

[16] Harrell FE (2006). Regression modeling strategies.

[17] Wang Y, Zheng F, Gao G (2018). MiR-548a-3p regulates inflammatory response via TLR4/NF-κB signaling pathway in rheumatoid arthritis. J Cell Biochem.

[18] Zhao X, Li R, Qiao M, Yan J, Sun Q (2018). MiR-548a-3p promotes keratinocyte proliferation targeting PPP3R1 after being induced by IL-22. Inflammation.

[19] Motaei J, Kerachian MA, Mousavi SA (2021). Circulating miR-455-3p, miR-5787, and miR-548a-3p as potential noninvasive biomarkers in the diagnosis of acute graft-versus-host disease: a validation study. Ann Hematol.

[20] Shim SM, Jung SY, Nam HY (2013). Network signatures of cellular immortalization in human lymphoblastoid cell lines. Biochem Biophys Res Commun.

[21] Herrmann MM, Barth S, Greve B, Schumann KM, Bartels A, Weissert R (2016). Identification of gene expression patterns crucially involved in experimental autoimmune encephalomyelitis and multiple sclerosis. Dis Model Mech.

[22] Nuñez-Borque E, Fernandez-Bravo S, Rodriguez Del Rio P (2021). Increased miR-21-3p and miR-487b-3p serum levels during anaphylactic reaction in food allergic children. Pediatr Allergy Immunol.

[23] Li X, Lou X, Xu S, Du J, Wu J (2020). Hypoxia inducible factor-1 (HIF-1α) reduced inflammation in spinal cord injury via miR-380-3p/ NLRP3 by Circ 0001723. Biol Res.

[24] Tetik Vardarlı A, Düzgün Z, Erdem C, Kaymaz BT, Eroglu Z, Çetintas VB (2018). Matrine induced G0/G1 arrest and apoptosis in human acute T-cell lymphoblastic leukemia (T-ALL) cells. Bosn J Basic Med Sci.

[25] Gnanakkumaar P, Murugesan R, Ahmed SSSJ (2019). Gene regulatory networks in peripheral mononuclear cells reveals critical regulatory modules and regulators of multiple sclerosis. Sci Rep.

[26] Ebrahimkhani S, Beadnall HN, Wang C (2020). Serum exosome microRNAs predict multiple sclerosis disease activity after fingolimod treatment. Mol Neurobiol.

[27] Fenoglio C, Riz MD, Pietroboni AM (2016). Effect of fingolimod treatment on circulating miR-15b, miR23a and miR-223 levels in patients with multiple sclerosis. J Neuroimmunol.

[28] Boffa G, Bruschi N, Cellerino M (2020). Fingolimod and dimethyl-fumarate-derived lymphopenia is not associated with short-term treatment response and risk of infections in a real-life MS population. CNS Drugs.

[29] Quirant-Sánchez B, Hervás-García JV, Teniente-Serra A (2018). Predicting therapeutic response to fingolimod treatment in multiple sclerosis patients. CNS Neurosci Ther.

