# Supplementary material for: miRNA 548a-3p as biomarker of NEDA-3 at 2 years in multiple sclerosis patients treated with fingolimod
Source: J Neuroinflammation. 2023 May 30;20:131. doi: 10.1186/s12974-023-02811-z (PMC10227972; doi:10.1186/s12974-023-02811-z)
Supplement: Supplementary file 2 — Additional file 2: Table S1. Univariate analysis of clinical variables in MS patients treated with Fingolimod regarding NEDA-3 at 2 years. Table S2. Previously reported roles of miRNA included in validation cohort. Figure S1. Exploratory functional analysis of the effect of miR-548a-3p mimic and inhibitors on T cell activation. [file 12974_2023_2811_MOESM2_ESM.docx]

**Additional file 2**

**TABLE S1. Univariate analysis of clinical variables in MS patients treated with Fingolimod regarding NEDA-3 at two years.**

|  | **ALL SAMPLES** | **NEDA 3** | **EDA** | **p value** |
| --- | --- | --- | --- | --- |
| **Discovery set** |  |  |  |  |
| No participants | 31 | 19 | 12 |  |
| Mean age | 40.71 (9.81) | 41.79 (10.17) | 39 (9.38) | 0.569 |
| Females | 71 | 73.7 | 66.7 | 0.989 |
| Mean EDSS | 1.52 (1.17) | 1.5 (1.07) | 1.54 (1.36) | 0.835 |
| Mean relapses | 0.65 (1.54) | 0.74 (1.85) | 0.5 (0.9) | 0.980 |
| Lymphocyte counts (cell/mm3) | 452.6 (249.7) | 458.9 (270.5) | 442.5 (224.1) | 0.98 |
| Lymphopenia 1 + 2 (%) | 19.4 | 26.3 | 8.3 | 0.36 |
| Lymphopenia 3 + 4 (%) | 67.7 | 63.2 | 75.0 | 0.7 |
| **Validation set** |  |  |  |  |
| No participants | 22 | 17 | 5 |  |
| Mean age | 44.36 (11.09) | 42.65 (10.89) | 50.18 (10.79) | 0.248 |
| Females | 81.8 | 82.4 | 80 | 1 |
| Mean EDSS | 1.82 (1.28) | 1.38 (1.11) | 3.3 (0.27) | 0.002** |
| Mean relapses | 0.45 (0.67) | 0.53 (0.72) | 0.2 (0.45) | 0.381 |
| Lymphocyte counts (cell/mm3) | 443.2 (176.3) | 415.9 (160.7) | 536.0 (214.3) | 0.21 |
| Lymphopenia 1 + 2 (%) | 31.8 | 29.4 | 40.0 | 1 |
| Lymphopenia 3 + 4 (%) | 63.6 | 70.6 | 40.0 | 0.31 |

**p<0.001

**TABLE S2. Previously reported roles of miRNA included in validation cohort.**

| **miRNA** | **Autoimmune condition** | **REF** |
| --- | --- | --- |
| miR-487b-3p | Anaphylactic reaction | ^22^ |
| miR-380-3p | Spinal cord injury inflammation | ^23^ |
| miR-376b-3p | T-cell lymphoblastic leukemia (T-ALL) cells | ^24^ |
| miR-301b | Multiple Sclerosis | ^25^ |
| miR-548a-3p | Rheumatoid arthritis and psoriasis | ^17–19^ |

**FIGURE S1. Exploratory functional analysis of the effect of miR-548a-3p mimic and inhibitors on T cell activation.**

**A.**


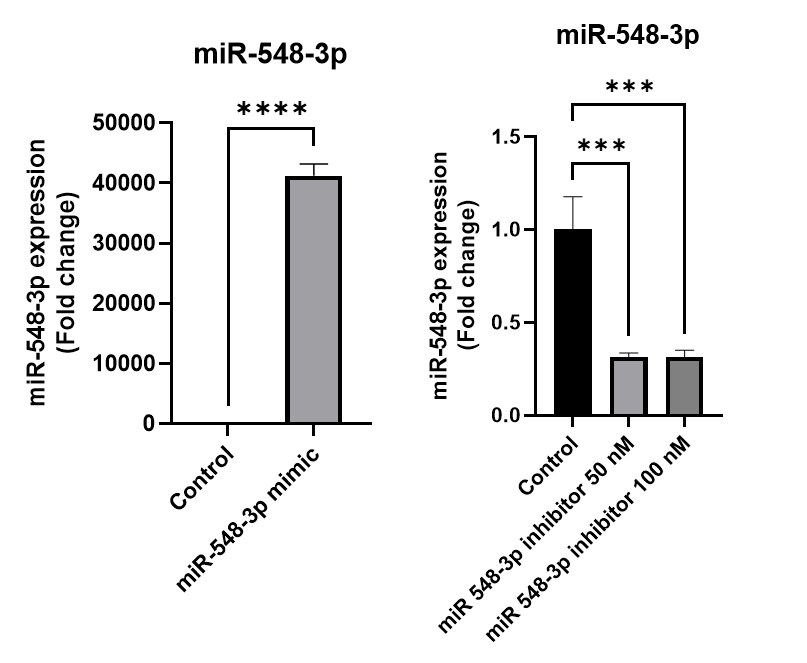

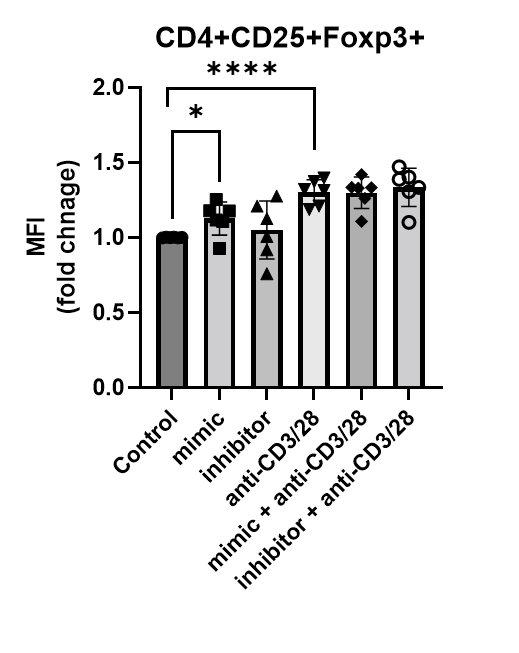


**B.**


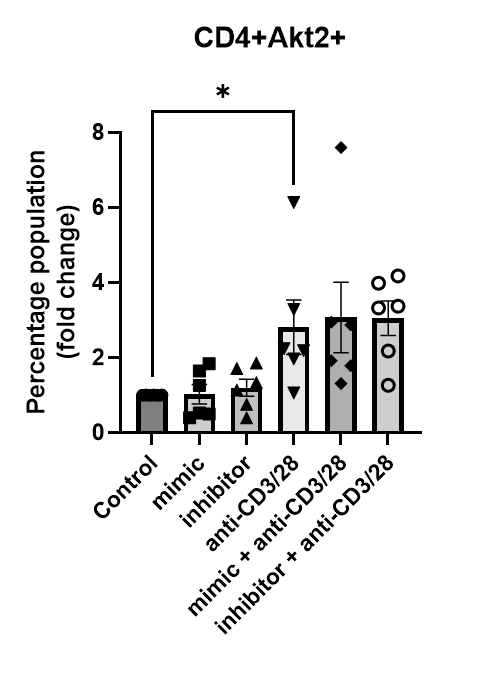


**C.**


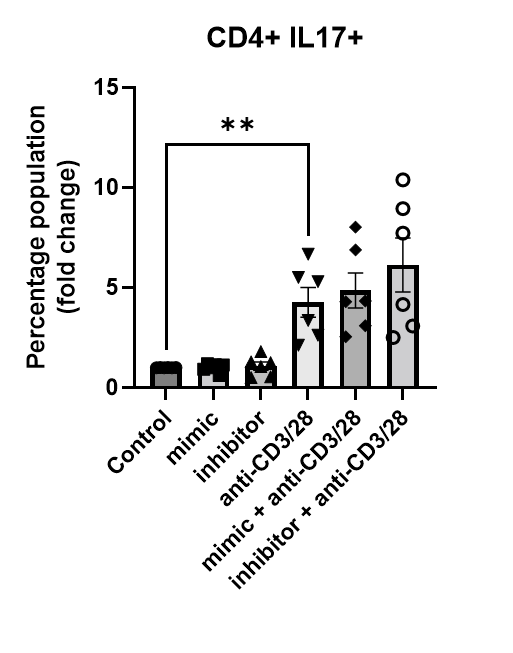


**D.**


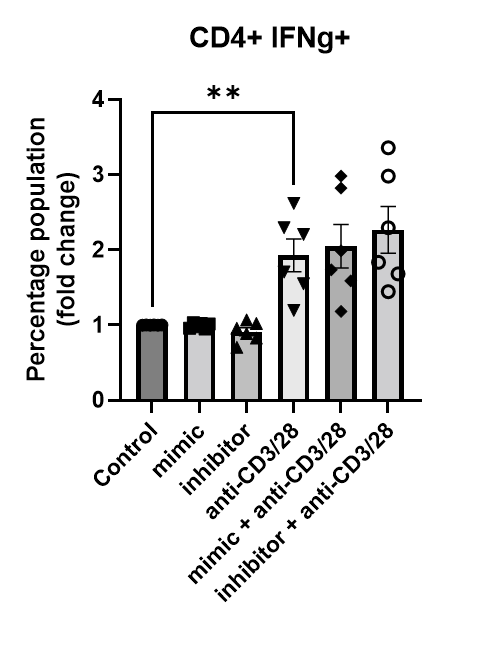


**E.**


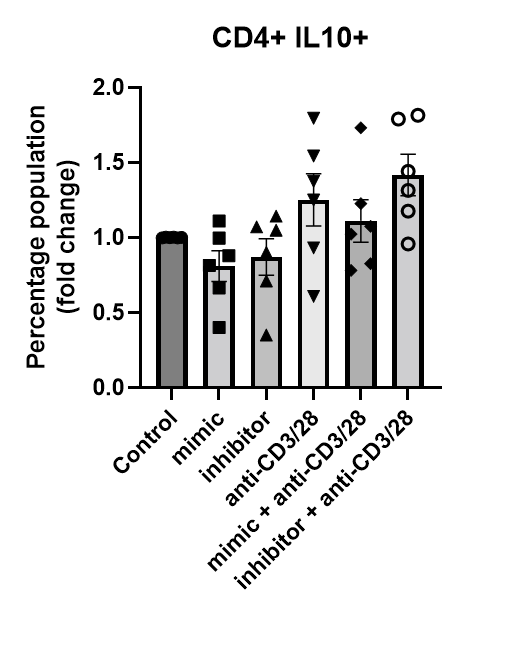


**F.**

(A) Expression of miR-548-3p post 24 h transfection of miR-548-3p mimic or inhibitor in pan-T cells, was analyzed by qPCR. (B) Flow cytometry analysis showing MFI of Treg cells indicated as CD4+CD25+Foxp3+. (C) (D) (E) and (F) Flow cytometry analysis showing expression of Akt2, IL17, IFNg and IL10, respectively, in CD4+ T-cells (indicated as % population). n=6. MFI: Mean Fluorescence Intensity; inhibitor: miR-548-3p inhibitor; mimic: miR-548-3p mimic; CD4+CD25+Foxp3: Treg population; anti-CD3/28: activated T reg population; Akt2: serine/threonine kinase 2; IL17: interleukin 17; IFNg: interferon Gamma; IL10: interleukin 10; *p<0.05; **p<0.01;***p<0.001; ****p<0.0001.
